# Expression of protocadherin-20 in mouse taste buds

**DOI:** 10.1038/s41598-020-58991-8

**Published:** 2020-02-06

**Authors:** Fumie Hirose, Shingo Takai, Ichiro Takahashi, Noriatsu Shigemura

**Affiliations:** 1 Section of Oral Neuroscience, Faculty of Dental Science, Kyushu University, Fukuoka, Japan; 2 Section of Orthodontics and Dentofacial Orthopedics, Division of Oral Health, Growth and Development, Faculty of Dental Science, Kyushu University, Fukuoka, Japan; 3 Division of Sensory Physiology, Research and Development Center for Five-Sense Devices Taste and Odor Sensing, Kyushu University, Fukuoka, Japan

**Keywords:** Taste receptors, Gustatory cortex

## Abstract

Taste information is detected by taste cells and then transmitted to the brain through the taste nerve fibers. According to our previous data, there may be specific coding of taste quality between taste cells and nerve fibers. However, the molecular mechanisms underlying this coding specificity remain unclear. The purpose of this study was to identify candidate molecules that may regulate the specific coding. GeneChip analysis of mRNA isolated from the mice taste papillae and taste ganglia revealed that 14 members of the cadherin superfamily, which are important regulators of synapse formation and plasticity, were expressed in both tissues. Among them, protocadherin-20 (Pcdh20) was highly expressed in a subset of taste bud cells, and co-expressed with taste receptor type 1 member 3 (T1R3, a marker of sweet- or umami-sensitive taste cells) but not gustducin or carbonic anhydrase-4 (markers of bitter/sweet- and sour-sensitive taste cells, respectively) in circumvallate papillae. Furthermore, Pcdh20 expression in taste cells occurred later than T1R3 expression during the morphogenesis of taste papillae. Thus, Pcdh20 may be involved in taste quality-specific connections between differentiated taste cells and their partner neurons, thereby acting as a molecular tag for the coding of sweet and/or umami taste.

## Introduction

Taste information is detected by taste bud cells located on the tongue, soft palate, larynx and epiglottis and then transmitted to the brain through the taste nerve fibers. Recent molecular studies have discovered candidate receptors for five basic tastes. These receptors are divided into two types: G protein-coupled receptors for sweet, bitter and umami tastes and channel-type receptors for salty and sour tastes. Receptors for sweet [taste receptor type 1 member 2 (T1R2) and T1R3], bitter [taste receptor type 2 (T2R)], salty [epithelial sodium channel (ENaC)] and sour (otopetrin-1) tastes are expressed in distinct subsets of cells in the taste buds^1–3^, which suggests that the coding of taste quality may occur at the level of taste cells. Moreover, a large number of taste cells respond to one of the five basic taste stimuli. The stimulation of taste cells may lead to the release of neurotransmitters that activate a particular population of gustatory nerve fibers. The response profiles of taste cells and gustatory nerve fibers are very similar, implying that many gustatory nerve fibers selectively innervate their corresponding type of taste cell^4–6^. It is noteworthy that although taste bud cells continuously regenerate with an average turnover of 5–20 days^7,8^, the specificity of the different taste qualities is maintained. This suggests that a system exists whereby taste quality-specific connections are made between newly developed taste receptor cells and matched gustatory nerve fibers. However, the underlying molecular mechanisms remain unclear.

The existence of specific cell surface identities would allow cells and neurons to discriminate one another and connect with their corresponding partners. Axon guidance molecules are key regulators of neural circuit assembly and function. Several candidate axon guidance molecules have been identified in taste tissues. Neurotrophin-4 and brain-derived neurotrophic factor were reported to promote geniculate ganglion (GG) neurite outgrowth^9^, while ephrin-B signaling was required for the innervation of lingual gustatory papillae^10^. However, these candidates are considered to regulate organ-level guidance of GG neurons to taste bud regions rather than single cell-level guidance of distinct neuronal populations coding a single taste quality to the corresponding taste receptor cells. A recent study demonstrated that semaphorin-7A (SEMA7A) and semaphorin-3A (SEMA3A) expressed in taste cells participated in axon guidance for their corresponding sweet and bitter taste nerve fibers, respectively^11^. However, after ablation of SEMA3A in taste bud cells by gene knockout, about 50% of GG neurons still responded to a single taste quality including bitter stimuli (singly tuned), implying that the taste quality wiring mechanism for bitter stimuli is not completely abolished by SEMA3A deletion from bitter taste-sensitive cells^12^. Therefore, an additional system may exist that directs bitter taste-sensitive cells toward matched bitter taste-responsive neurons. In addition, guidance molecules specific for umami, salty and sour tastes have not been identified yet.

Cadherins are a superfamily of more than 100 transmembrane glycoproteins that act as cell-cell adhesion molecules. Cadherins regulate morphogenesis in a variety of organs during development and are also involved in synaptogenesis in the vertebrate central nervous system^13^. The diversity of molecular structures within this family imparts neuronal cell types with specific surface identities that enables homophilic interactions to occur between cells^14^. For instance, in the olfactory epithelium, E-cadherin (Cdh1) mRNA was detected only in supporting cells, whereas N-cadherin (Cdh2) mRNA was detected in both olfactory cells and supporting cells. When cells expressing either E- or N-cadherin were randomly mixed, the cells segregated from each other after culture for 72 hours^15^. In the retina, Cdh8 and Cdh9 are each expressed selectively by one of two bipolar cell types, and perturbation of their expression patterns led to mismatched wiring between the cells and their corresponding neurons, which in turn resulted in distinct defects in visually-evoked responses^16^.

Therefore, we hypothesized that the expression of distinct cadherins in both taste bud cells and taste ganglion neurons might be involved in taste quality-specific coding mechanisms during continuous taste cell turnover. To explore this possibility, we utilized GeneChip analysis, reverse transcription polymerase chain reaction (RT-PCR), *in situ* hybridization (ISH) and double-staining immunohistochemistry to investigate the expression patterns of cadherin candidates in the taste buds, taste ganglia and non-taste trigeminal ganglion (TG) of infant and adult mice.

## Results

### GeneChip analysis identified 14 cadherin superfamily candidates expressed in both taste buds and taste ganglia

**Figure 1:**
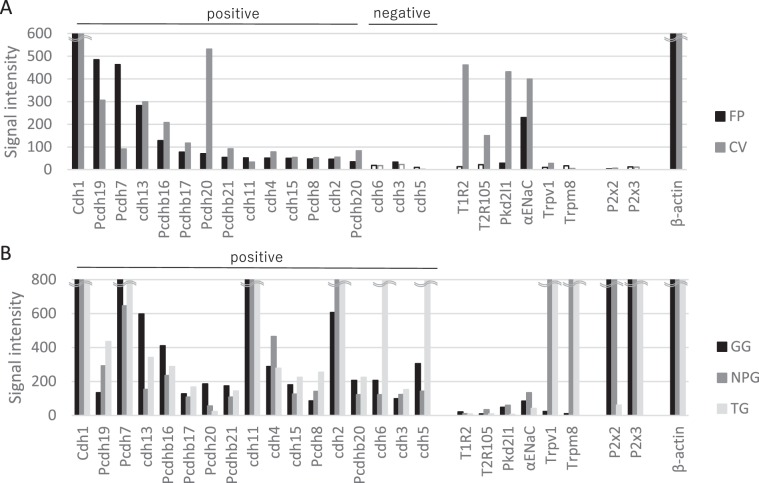
GeneChip analysis of the mRNA expressions of cadherins and protocadherins in mouse taste buds (circumvallate and fungiform papillae), cranial taste ganglia (geniculate ganglion and nodose-petrosal ganglion complex) and non-taste trigeminal ganglion. GeneChip analysis identified 14 candidates of the cadherin superfamily that were positively expressed in both taste buds and taste ganglia (refer to Supplementary Table S1). (**A**) Bar graph showing the microarray signal intensity for each gene in the fungiform papillae (FP, black bars) and circumvallate papillae (CV, gray bars). ‘Positive’ and ‘negative’ indicate the expression level suggested by GeneChip analysis. (**B**) Microarray-detected expression levels for each gene in the geniculate ganglion (GG, black bars), nodose-petrosal ganglion complex (NPG, gray bars) and trigeminal ganglion (TG, light gray bars). ‘Positive’ and ‘negative’ indicate the gene expression level evaluated by GeneChip analysis, and Cdh15 and Pcdh8 were added as positively expressed genes. Cdh, cadherin; ENaC, epithelial sodium channel; P2x2/3, purinergic receptor P2x2/3; Pcdh, protocadherin; Pkd2L1, polycystin 2 like 1; T1R2, taste receptor type 1 member 2; T2R105, taste receptor type 2 member 105; Trpv1, transient receptor potential cation channel subfamily V member 1; Trpm5/8, transient receptor potential cation channel subfamily M member 5/8.

To identify candidate guidance molecules that regulate specific synapse formation, we performed DNA microarray analysis on the taste buds [circumvallate papillae (CV) and fungiform papillae (FP)], cranial taste ganglia [GG and nodose-petrosal ganglion complex (NPG)] and non-taste TG of B6 mice. The GeneChip expression analysis revealed that mRNAs for 14 of the 59 cadherin superfamily genes listed in the DNA microarray data (cadherins Cdh1, Cdh2, Cdh4, Cdh11, Cdh13 and Cdh15; and protocadherins Pcdh7, Pcdh8, Pcdh19, Pcdh20, Pcdhb16, Pcdhb17, Pcdhb20 and Pcdhb21) were positively expressed in both taste buds and taste ganglia (Fig. 1A,B, Supplementary Table S1). Among these 14 genes, only Pcdh20 was not detected in the non-taste TG (Fig. 1B). mRNA markers for sweet taste-sensitive cells (T1R2), bitter taste-sensitive cells (T2R105), sour taste-sensitive cells [polycystin 2 like 1 (Pkd2L1)] and salty taste-sensitive cells (αENaC) were predominantly expressed in the taste buds but not in cranial ganglia, whereas mRNA markers for taste-responsive neurons (purinergic P2x2 and P2x3 receptors) were detected in the cranial ganglia but not in the taste buds. In addition, mRNA markers for somatosensory neurons responding to hot and cold stimuli [transient receptor potential cation channel subfamily V member 1 (Trpv1) and transient receptor potential cation channel subfamily M member 8 (Trpm8)] were preferentially expressed in the NPG and TG but not in the taste buds or GG. The expression patterns of these molecular markers are consistent with previous studies^1,3,17^.

### RT-PCR revealed Pcdh20 mRNA expression in both taste buds and taste ganglia

**Figure 2:**
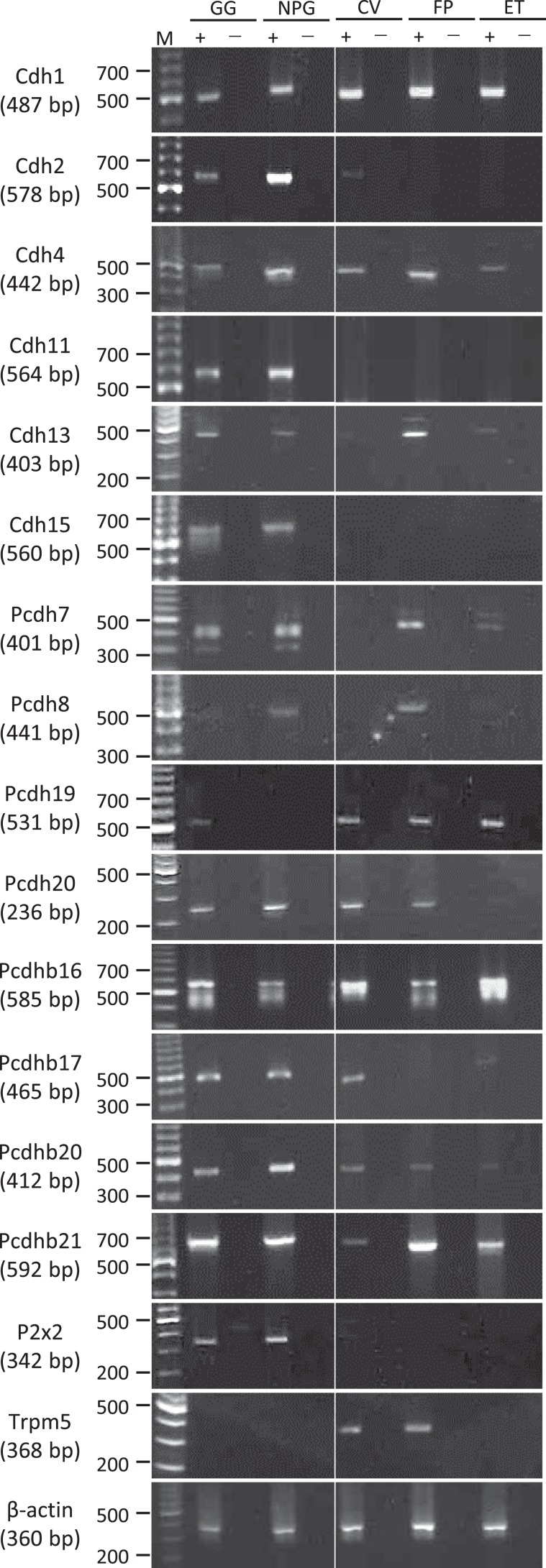
Protocadherin-20 (Pcdh20) mRNA is expressed in mouse taste buds and taste ganglia but not in non-taste tongue epithelium. Reverse transcription-polymerase chain reaction (RT-PCR) amplification of the mRNAs of 14 members of the cadherin superfamily detected by GeneChip analysis, the purinergic P2x2 receptor (P2x2), the transient receptor potential channel M5 (Trpm5) and β-actin from the geniculate ganglion (GG), nodose-petrosal ganglion complex (NPG), circumvallate papillae (CV), fungiform papillae (FP) and tongue epithelium devoid of taste cells (ET). RT+ and RT− conditions are, respectively, with and without reverse transcriptase. M (bp): 100 bp marker ladder. All experiments were performed as follows: 95 °C for 15 min (1 cycle); 94 °C for 30 s, 55 °C for 30 s and 72 °C for 36 s (35 cycles).

In order to validate the data obtained from the GeneChip analysis, we performed RT-PCR experiments to evaluate the mRNA expressions of 14 cadherin members in the CV, FP, GG, NPG and non-taste epithelial tissue (ET) of B6 mice (Fig. 2). A PCR band of the correct size (236 bp) for Pcdh20 mRNA was clearly detected in the four taste tissues (FP, CV, GG and NPG) but not in ET devoid of taste buds, whereas the other 13 cadherin members were expressed not only in taste tissues but also in ET (Cdh1, 4, 13, 7, Pcdh19, b16, b20, b21), either not expressed in both FP and CV (Cdh2, 11, 15, Pcdh8, b17). These results suggest that Pcdh20 may be a taste tissue-specific molecular tag. As positive controls, RT-PCR products for the type II taste cell marker Trpm5 (368 bp) were found in both FP and CV taste buds but not in ET, GG or NPG, and the taste neuron marker P2x2 (342 bp) was found in both the cranial GG and NPG but not in FP, CV or ET. β-actin mRNA (360 bp) was detected in all tissues. All control experiments in which the reverse transcriptase enzyme was omitted (RT-) yielded negative results.

### ISH demonstrated that Pcdh20 mRNA is expressed in a subset of taste bud cells

**Figure 3:**
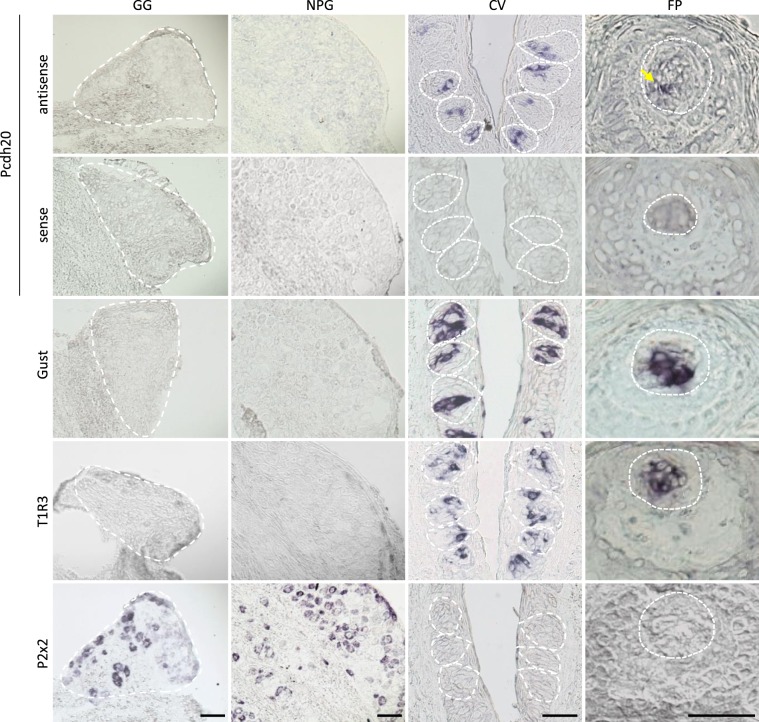
Protocadherin-20 (Pcdh20) is expressed in a subset of mouse taste bud cells but not in taste ganglion neurons. *In situ* hybridization analysis of Pcdh20, taste receptor type 1 member 3 (T1R3), gustducin (Gust) and the purinergic P2x2 receptor (P2x2) in circumvallate papillae (CV), fungiform papillae (FP), geniculate ganglion (GG) and nodose-petrosal ganglion complex (NPG) of B6 mice. The sense probes served as a negative control. Dotted lines indicate the outlines of taste buds. Arrow denotes Pcdh20-positive cells. Scale bars, 50 µm.

Next, we examined the expression of Pcdh20 in mouse taste bud cells and taste ganglion neurons using ISH analysis. Pcdh20 mRNA was detected in a subset of taste bud cells in both the FP and CV but not in surrounding epithelial cells (Fig. 3). We did not observe clear signals for Pcdh20 mRNA in either the GG or NPG, suggesting that the Pcdh20 mRNA expression level in sensory neurons may be low compared with that in taste bud cells. This result appears consistent with the data from GeneChip analysis (Fig. 1). As controls, signals for T1R3 (a sweet/umami taste cell marker) and gustducin (a bitter/sweet taste related G protein^1^) were detected in both FP and CV taste buds but not in GG or NPG. Signals for the taste neuron marker, P2x2, were observed in both the GG and NPG but not in FP or CV. Control hybridization using a Pcdh20 sense probe was negative. These results, together with the data from the RT-PCR experiments, suggest that Pcdh20 mRNA is more strongly expressed in a subset of mouse taste bud cells than in cranial ganglia.

### Pcdh20 is co-expressed with T1R3 in taste receptor cells

**Figure 4:**
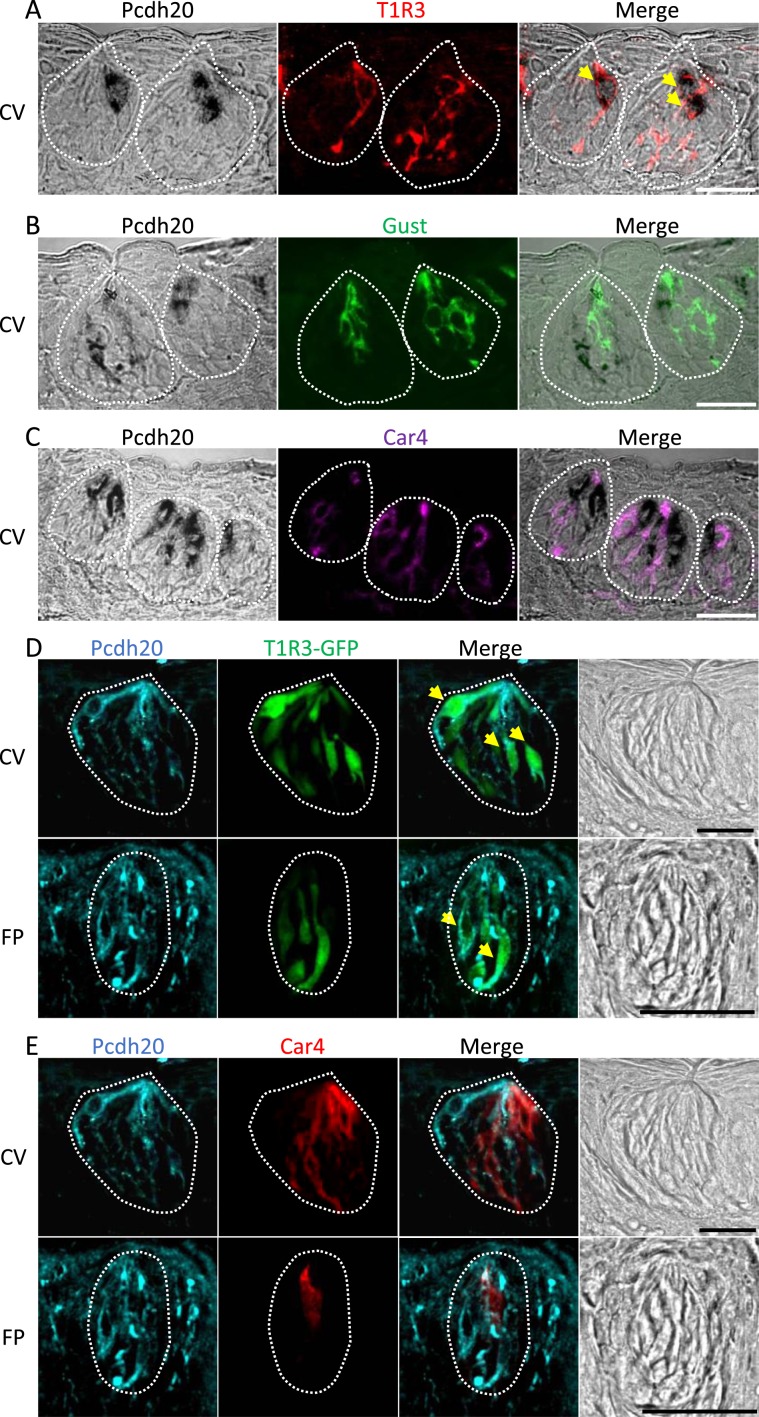
Protocadherin-20 (Pcdh20) is co-expressed with taste receptor family 1 member 3 (T1R3) in mouse taste bud cells of circumvallate papillae and fungiform papillae. (**A**) A combined analysis using immunostaining and *in situ* hybridization demonstrated co-expression of Pcdh20 with T1R3 (a sweet or umami receptor component). (**B**) Pcdh20 was not co-expressed with gustducin (Gust, a G protein mediating bitter and sweet taste transduction). (**C**) Pcdh20 was not co-expressed with carbonic anhydrase-4 (Car4, a sour taste-sensitive cell marker). Signals obtained by *in situ* hybridization for Pcdh20 are shown in black, and those obtained by immunostaining for T1R3, Gust and Car4 are shown in red, green and purple, respectively. (**D,E**) Double-detection analysis using T1R3-GFP mice demonstrated that some of the immunoreactivity for Pcdh20 protein was observed in cells expressing T1R3-GFP signals, but immunoreactivity for Pcdh20 was not detected in Car4-positive cells in the circumvallate papillae (CV) and fungiform papillae (FP). Arrows denote Pcdh20 + T1R3 double-positive cells. Dotted lines indicate the outlines of taste buds. Scale bars, 25 µm.

**Figure 5:**
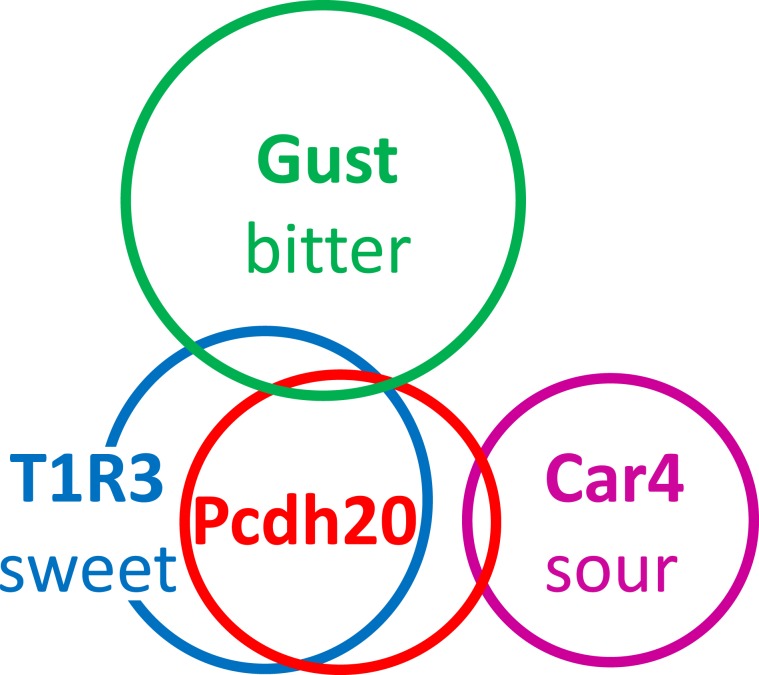
Summary of the patterns of co-expression between protocadherin-20 (Pcdh20) and taste cell markers in taste cells of the circumvallate papillae. The areas of the circles indicate the numbers of taste bud cells expressing Pcdh20, taste receptor type 1 member 3 (T1R3, a sweet or umami taste cell marker), gustducin (Gust, a G protein mediating mainly bitter taste transduction in mouse circumvallate papillae^19^) and carbonic anhydrase-4 (Car4, a sour taste cell marker) in the circumvallate papillae of mice. The overlapped area indicates the co-expression. This diagram is based on Table 1.

To further assess which taste bud cell types express Pcdh20, we performed immunostaining in combination with ISH. In CV, a subset of Pcdh20 mRNA-positive cells expressed the sweet/ umami receptor component, T1R3 (T1R3/Pcdh20: 71.7%), but very few Pcdh20-positive cells expressed the bitter/sweet taste related G protein, gustducin (gustducin/Pcdh20: 8.0%) or the sour taste cell marker, carbonic anhydrase-4 (Car4)^18^ (Car4/Pcdh20: 7.8%) (Fig. 4A–C). The inverse co-expression ratios (Pcdh20/markers) are shown in Table 1. A summary of the expression patterns of Pcdh20 and taste cell markers in CV is shown in Fig. 5. Furthermore, some immunoreactivity for Pcdh20 protein was observed in cells expressing T1R3-GFP signals but not in Car4-positive cells in both CV and FP (Fig. 4D,E).

**Table 1:** Co-expression ratios for protocadherin-20 (Pcdh20) and taste cell markers in mouse circumvallate papillae.

T1R3/Pcdh20	71.7%	(94/131, n = 92)	Pcdh20/T1R3	59.9%	(94/168, n = 92)
Gust/Pcdh20	8.00%	(15/188, n = 121)	Pcdh20/Gust	6.20%	(15/246, n = 121)
Car4/Pcdh20	7.80%	(9/115, n = 84)	Pcdh20/Car4	7.50%	(9/120, n = 84)

The data in parentheses show the number of cells positive for both gene 1 and gene 2/the number of cells positive for gene 2, and n = number of taste buds examined. Car4, carbonic anhydrase-4; Gust, gustducin; Pcdh20, protocadherin-20; T1R3, taste receptor type 1 member 3.

### Pcdh20 expression in taste cells occurs later than T1R3 expression during taste bud development

**Figure 6:**
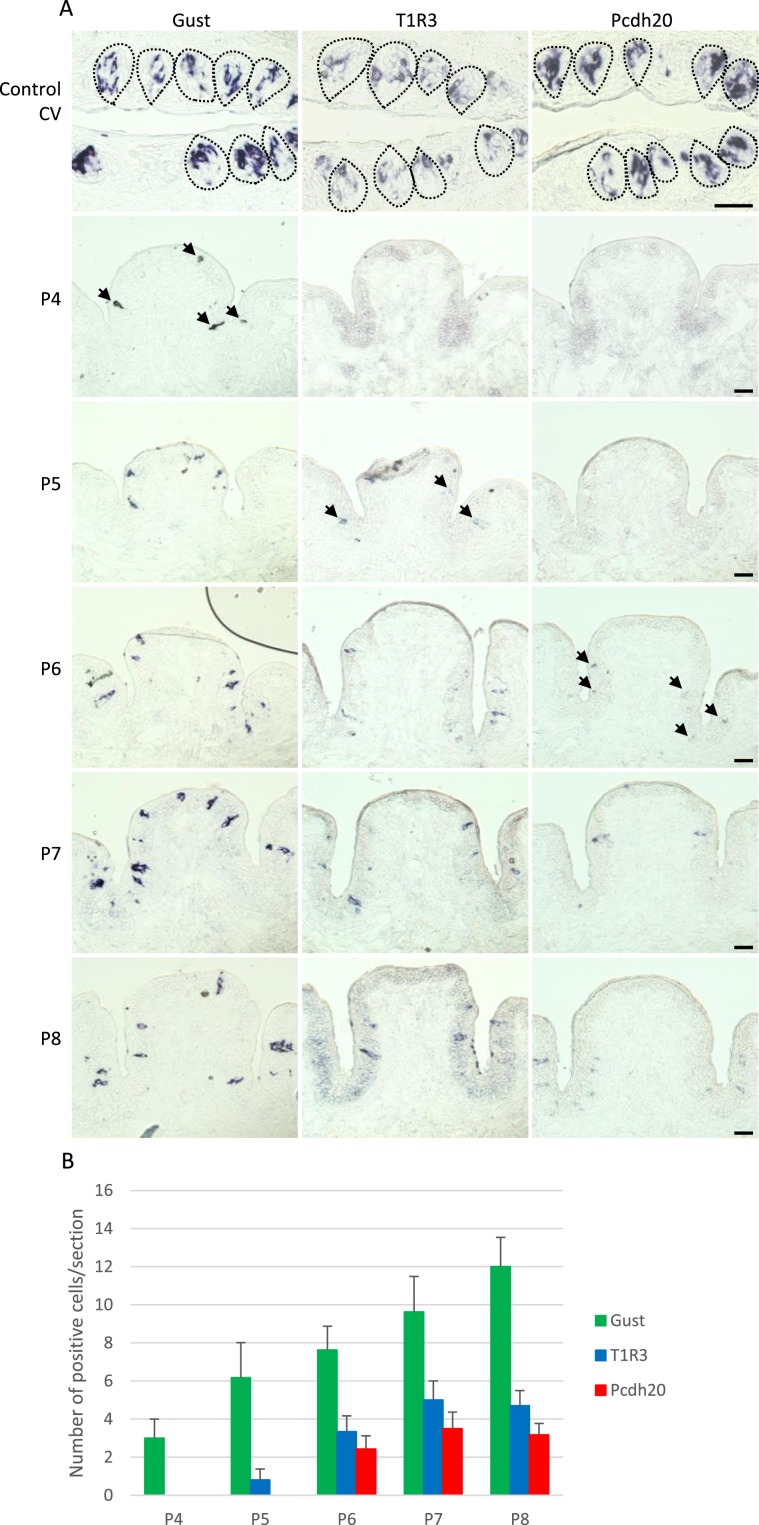
Protocadherin-20 (Pcdh20) expression in taste cells occurs later than taste receptor type 1 member 3 (T1R3) expression during the morphogenesis of taste papillae. (**A**) *In situ* hybridization analysis using tissues from infant mice revealed that Pcdh20 mRNA was first detected in the developing mouse circumvallate papillae (CV) at postnatal day 6 (P6), whereas T1R3 and gustducin (Gust) mRNAs were observed from postnatal day 4–5 (P4–5), i.e. before the expression of Pcdh20. Arrows denote Pcdh20, T1R3 and gustducin-positive cells. Dotted lines indicate the outlines of taste buds. Scale bars, 50 µm. (**B**) The temporal changes in the number of Gust (green), T1R3 (blue) or Pcdh20 (red) positive cells during taste papilla development. Each score is expressed as mean ± standard error of the mean per section [n = 3–10 sections obtained from 1 (P4) to 4 mice (P5–8) per postnatal day, in independent preparations].

We also performed ISH analyses to examine the temporal change of the Pcdh20 mRNA expression patterns and its levels in taste cells during postnatal development. Pcdh20 mRNA had appeared in the surface epithelial cells of the CV area but not in the mesenchymal tissue at postnatal day 6 (Fig. 6A,B). Gustducin and T1R3 mRNAs were detected in the surface epithelial cells of the CV from postnatal day 4 and 5, respectively (Fig. 6A,B). These results suggest that the expression of Pcdh20 mRNA in taste cells occurs later than that of gustducin and T1R3 during taste bud development, implying that the expression of Pcdh20 may be regulated so as to occur in differentiated taste receptor cells but not in undifferentiated cells in the taste buds.

## Discussion

In the present study, GeneChip and RT-PCR analyses revealed that the mRNAs for several cadherins (Cdh1, Cdh2, Cdh4, Cdh11, Cdh13 and Cdh15) and protocadherins (Pcdh7, Pcdh8, Pcdh19, Pcdh20, Pcdhb16, Pcdhb17, Pcdhb20 and Pcdhb21) were expressed in mouse taste papillae (FP and CV) and cranial ganglia (GG and NPG). Among these cadherin superfamily members, Pcdh20 mRNA was more highly expressed in the taste buds than in the taste ganglia and was not detected in the non-taste TG and surrounding tongue epithelial cells. In CV, double detection with ISH and immunohistochemistry demonstrated that Pcdh20 was co-expressed with T1R3 (a sweet- or umami-sensitive taste cell marker) but not with gustducin (a bitter/sweet taste related G protein) or Car4 (a sour-sensitive taste cell marker). During the postnatal period, Pcdh20 mRNA was detected from postnatal day 6, later than the initial expression of T1R3 at postnatal day 5. In mouse CV, approximately 70% and 10% of gustducin positive cells co-expressed bitter taste receptor T2Rs^19^ and sweet taste receptor component T1R2^20,21^, respectively, suggesting that gustducin has a prominent role in bitter taste transduction in the posterior region of the tongue. These results suggest that Pcdh20 may be a molecular tag for sweet or umami taste coding, facilitating taste quality-specific connections between differentiated taste cells and their partner neurons.

T1R3 is known to function as sweet or umami taste receptors in combination with T1R2 or T1R1, respectively^3^. Co-expression analysis among T1Rs in taste tissues showed that, in CV, 92% of T1R3-positive cells co-expressed T1R2, and about 50% of the T1R2 + T1R3 double positive cells co-expressed T1R1, while in FP, 48% and 65% of T1R3-positive cells co-expressed T1R2 and T1R1 respectively, and about 50% of the T1R2 + T1R3 double positive cells co-expressed T1R1^20^. Another study reported that 33% of T1R1-positive cells co-expressed both T1R2 and T1R3, and about 25% of the T1R1-positive cells co-expressed only T1R3 in mouse FP^22^. T1R3-knockout mice exhibited significantly reduced glossopharyngeal nerve responses to sweet but not to umami tastants, which innervates CV^23^. In contrast, in the chorda tympani nerve innervating FP, such reduced responses were observed not only for sweet but also umami tastants. These results suggest that T1R3 may underlie only sweet responses in CV, and both sweet and umami responses in FP. Thus, Pcdh20-positive cells with T1R3 might be involved in a role of sweet taste coding in CV, and of sweet and/or umami taste codings in FP.

Protocadherins, which represent the largest subgroup of the cadherin superfamily, are subdivided into clustered (over 50 members) and non-clustered (about 20 members) protocadherins. The clustered protocadherins are organized into three gene clusters designated α, β and γ^13^. Pcdhb16, Pcdhb17, Pcdhb20 and Pcdhb21, which were detected in this study, are grouped in the β-cluster, whereas Pcdh7, Pcdh8, Pcdh19 and Pcdh20 are non-clustered protocadherins. Experiments in non-neuronal cells suggest that clustered protocadherins mediate homophilic interactions in which the adhesion molecule on the surface of one cell binds to the same molecule on the surface of another cell^24^. Clustered protocadherins in the nervous system have been suggested to play roles in the formation and maintenance of synaptic connections, whereas the role of non-clustered protocadherins in connectivity is not well understood^25^. A study examining the spatiotemporal distribution of mRNAs for 12 non-clustered protocadherins in rat brain revealed that the expression of each protocadherin was restricted to distinct brain regions. For instance, Pcdh7 and Pcdh20 mRNAs were predominantly expressed in the somatosensory and visual cortices, whereas Pcdh11 and Pcdh17 mRNAs were preferentially expressed in the motor and auditory cortices, suggesting that protocadherins might act as molecular tags for the recognition of distinct neural circuits^26^. Regarding Pcdh20, it has been reported that Pcdh20 immunoreactivity is higher in newly differentiated olfactory bulb sensory neurons than in other brain regions (such as the hippocampus, cerebral cortex, cerebellum and spinal cord) but is down-regulated in the adult olfactory system with some gender-based differences in expression levels^27^. A genome-wide association study in an Italian cohort suggested Pcdh20 as a candidate affecting hearing ability and loss, and RT-PCR and RNA-seq analyses demonstrated that Pcdh20 is expressed in the mouse inner ear, especially in utricle hair cells but not in surrounding cells^28^. These findings suggest that Pcdh20 may be a common neural guidance molecule for various sensory systems and function in the taste sensory system as a specific molecular tag for homophilic interactions, whereby Pcdh20 on the surface of taste cells binds to the same molecule on the surface of taste ganglion neurons.

During the development of CV, Pcdh20 was first detected in a distinct subset of epithelial cells at postnatal day 6, whereas both gustducin and T1R3 were expressed (albeit sparsely) from postnatal day 4, i.e. prior to the expression of Pcdh20. The temporal expression pattern of gustducin is consistent with a previous study showing that gustducin and/or T1R2 positive cells were detected from postnatal day 2.5–4.5 in mouse CV^29^. These results raise the possibility that Pcdh20 expression may be induced in differentiated taste receptor cells expressing T1R3. A previous electrophysiological study examining the recovery of NaCl responses in regenerating mouse chorda tympani after crush injury to the nerve revealed that a response sensitive to amiloride (a diuretic) reappeared at 4 weeks after the nerve crush. ENaC is composed of three subunits (α, β and γ) and considered to mediate the amiloride-sensitive response to NaCl in mouse taste cells. All three ENaC subunits were expressed in a subset of taste bud cells at 2 weeks after crush injury to the chorda tympani, and the mean number of ENaC-positive cells per taste bud had recovered to the control level at 3 weeks, implying that differentiation of taste cells into amiloride-sensitive responsive cells might occur prior to synapse formation^30^. Similarly, the differentiation of taste cells expressing T1R3 may occur prior to the recovery of the responsiveness of the chorda tympani to sweet taste stimuli^31^. These results suggest that Pcdh20 may function as a molecular tag that guides the connection of differentiated sweet or umami taste receptor cells to their corresponding nerve fibers, which would help to maintain individually-tuned taste responsiveness during the continuous turnover of taste bud cells throughout life.

Very recently, single-cell RNA-seq analysis of 800 GG neurons expressing P2x3, a receptor for the neurotransmitter (ATP) released from taste cells, identified five molecular markers for different neural populations mediating each of the five basic taste qualities: Cdh4 for umami, Cdh13 for bitter, early growth response-2 for salty, Spondin-1 for sweet and proenkephalin for sour^32^. Cdh4 and Cdh13 knockout mice showed a lack of behavioral preference for umami and no avoidance of bitter tasting solutions, respectively, without any changes in the preferences to the other taste qualities. In our study, Cdh4 and Cdh13 were detected not only in taste ganglion neurons (GG and NPG) but also in taste bud cells, suggesting that Cdh4 and Cdh13 may participate in homophilic interactions between distinct taste neurons and corresponding taste cells. Other cadherins (Cdh1, Cdh2, Cdh11, Cdh15, Pcdh7, Pcdh8, Pcdh19, Pcdhb16, Pcdhb17, Pcdhb20 and Pcdhb21) may also be involved in taste quality-specific connections either via expression of the individual cadherin or via multiple expression of a repertoire of cadherins.

In this study, we investigated the mRNA and protein expression of only Pcdh20 in taste buds and ganglia. Further research involving gene knockout or knockdown techniques will be needed to determine whether Pcdh20 and other cadherins are involved in taste-specific coding.

## Methods

All experiments and procedures were conducted in accordance with the National Institutes of Health *Guide for the Care and Use of Laboratory Animals* and approved by the committee for Laboratory Animal Care and Use at Kyushu University, Japan (approval no. A19-003).

### Animals

The experimental subjects were adult male and female C57BL/6NCrj (B6) mice (age, 8–16 weeks; weight, 20–32 g; Charles River, Tokyo, Japan) and their littermates (postnatal day 4–8) as well as transgenic T1R3-GFP mice co-expressing taste receptor type 1 member 3 (T1R3) and green fluorescent protein (GFP)^33^. All mice were housed in a constant room temperature of 24 ± 1 °C under a 12-hour light and 12-hour dark cycle (lights on at 0800) and given access to food and water *ad libitum*.

### DNA microarray analysis

Taste tissues and cranial ganglia were collected from B6 mice under pentobarbital anesthesia (50–60 mg/kg body weight). The trachea of each mouse was cannulated, and then the mouse was fixed in the supine position with the head in a holder. The GG, NPG and TG were dissected. Mouse individual taste buds were isolated from FP and CV in the peeled tongue epithelium by aspiration with a transfer pipette. Total RNAs were purified from the collected tissue samples using the RNeasy Plus Micro kit (Qiagen, Stanford, CA, USA). The GeneChip Mouse Genome 430 2.0 Array (Affymetrix, Santa Clara, CA, USA) was used for microarray analysis. RNA quality control, total RNA labeling, microarray hybridization and scanning were performed in accordance with the Affymetrix GeneChip Expression Analysis Technical Manual (www.affymetrix.com).

### RT-PCR

RT-PCR was conducted as described previously^34–36^. Taste buds were isolated from the FP and CV of each mouse by using a transfer pipette, and pooled. The RNeasy Plus Micro kit (Qiagen) was used to purify RNAs from 30 taste buds from the FP and CV respectively, or a 1 mm × 1 mm block of epithelial tissue without taste buds. cDNAs were generated by RT [oligo(dT)12–18 primer] with the superscript pre-amplification system (Invitrogen, Carlsbad, CA, USA). The primer sequences are shown in Table 2. To control for signals from genomic DNA, purified RNA samples were treated in parallel with or without reverse transcriptase. PCR reaction was carried out under the following condition: 95 °C for 15 min (1 cycle); 94 °C for 30 s, 55 °C for 30 s and 72 °C for 36 s (35 cycles). Each 20 μL of PCR solution was comprised of 0.5 U of TaqDNA polymerase (TaKaRa Ex TaqHS; Takara Bio, Kusatsu, Japan), 2 μL of 10× PCR buffer containing 20 mM Mg^2+^, 0.2 mM of each dNTP and 0.6 μM of each primer pair. The resulting amplification products were visualized in a 2% agarose gel with 0.5 μg/mL ethidium bromide. β-actin was used as the internal control.

**Table 2:** Nucleotide sequences for the primers used in RT-PCR and *in situ* hybridization.

Gene	Accession no.	Analysis	Forward	Reverse	Region
Cdh1	NM_009864.3	RT-PCR	AGCCATTGCCAAGTACATCC	AGGCACTTGACCCTGATACG	820–1306
Cdh2	NM_007664.5	RT-PCR	CGTGAATGGGCAGATCACTAC	TGAGAACAAGGATCAGCAGG	2496–3073
Cdh4	NM_009867.3	RT-PCR	GCCCTTCATCCCCACTACAG	GCTGAGGTCGTAGTCTTGGTC	2135–2576
Cdh11	NM_009866.5	RT-PCR	AAACTGCCTGGCTCAACATC	CAAACAGCACAACGATGACC	1855–2418
Cdh13	NM_019707.5	RT-PCR	CCCTATCTGCCATGCAAAACG	GAAGGTCAAGTTTAGGACAGGC	6–408
Cdh15	NM_007662.2	RT-PCR	TTGGACTTGGGTGGCTCTAC	ATCTTGAACTGCCCATCAGG	484–1043
Pcdh7	NM_001122758.2	RT-PCR	AAGTACAGCAAACAGCCATTTCG	TCCTGGCTTCCTCGTTCATC	4184–4584
Pcdh8	NM_021543.4	RT-PCR	ATGAAACCGCCAGGGGAAC	TACACGCCCACAGTCCACTC	2602–3042
Pcdh19	NM_001105245.1	RT-PCR	AGTGTCTGCATTGCATCTCG	TCTCCTCATGCCCACTATCC	3933–4463
Pcdh20	NM_178685	RT-PCR	GGGGCAAGCATCAAAACACATT	AAACACCAAGCAAGCAAGTAGAGATT	3604–3839
		ISH	CCTGCCGCATGTTTTTCTG	TTGGGTGCGTTGTTTTCTTC	247–1776
Pcdhb16	NM_053141.3	RT-PCR	CATCTGGTGGATGTCAGTGG	GAGCCCCTCGTTCTCCTATC	2430–3014
Pcdhb17	NM_053142.3	RT-PCR	GCTGGTGCTGGAGAAAGAAC	ATCACTTCTGGGGCATTGTC	717–1181
Pcdhb20	NM_053145.2	RT-PCR	TATACCCGGAGCTGGTTCTG	AGACCTCCACCATCTGTTGC	728–1139
Pcdhb21	NM_053146.2	RT-PCR	TAAGCCCCAACCCCTATTTC	AAAACAGCGACCACCATCTC	677–1268
P2x2	NM_153400	RT-PCR	CATCATCAATCTGGCCACTG	TCAAGAGTGTCCACCACCTG	1109–1450
		ISH	TGCACGTTTGATCAGGACTC	TCTGTTGGGAAAGGAAATGG	759–1765
Trpm5	NM_020277	RT-PCR	CAGGAGGCACCTACTCAAGC	ATCCAGCTGTCCAGTCCAAG	3458–3825
β-actin	NM_007393	RT-PCR	GGTTCCGATGCCCTGAGGCTC	ACTTGCGGTGCACGATGGAGG	840–1199
Gustducin	NM_001081143	ISH	AGATGGGAAGTGGAATTAGTTCAGA	GCTCAGAAGAGCCCACAGTCTTTGA	108–1174
T1R3	NM_031872	ISH	TGCTGCTATGACTGCGTGGAC	AAGAAGCACATAGCACTTGGG	1583–2488

Cdh, cadherin; ISH, *in situ* hybridization; P2x2, purinergic receptor P2x2; Pcdh, protocadherin; RT-PCR, reverse transcription polymerase chain reaction; T1R3, taste receptor type 1 member 3; Trpm5, transient receptor potential cation channel subfamily M member 5.

### ISH

RNA probes were prepared for ISH as previously described^30,34,37,38^. Primer sequences for ISH are listed in Table 2. RT-PCR products were purified and cloned into the pGEM T-Easy vector (Promega, Madison, WI, USA), confirmed by DNA sequencing and digested with appropriate restriction enzymes. Digoxigenin-UTP-labeled antisense and sense RNA probes were prepared by *in vitro* transcription using the SP6/T7 RNA polymerase Kit (Roche, Mannheim, Germany). Frozen blocks of the dissected tongue and cranial ganglia of B6 mice were embedded in optimal cutting temperature (OCT) compound (Sakura Finetechnical, Tokyo, Japan) and sliced into serial sections (6-μm thickness), which were placed on silane-coated glass slides. The cryosections were fixed in 4% paraformaldehyde (PFA) in phosphate-buffered saline (PBS) for 10 min at room temperature, washed with 5× standard saline citrate (SSC) for 15 min at room temperature, incubated in prehybridization solution (5 × SSC/50% formamide) for 1 h at room temperature, and then hybridized in a hybridization buffer containing 50% formamide, 5 × SSC, 5 × Denhardt’s solution, 250 μg/mL denatured baker’s yeast tRNA, 500 μg/mL denatured salmon testis DNA, 1 mM dithiothreitol and 20–200 ng/mL antisense RNA probe for 18 h at 60 °C. Subsequently, the sections were washed with 5 × SSC/50% formamide two times for 5 min and with 0.2 × SSC/50% formamide two times for 60 min at 65 °C. The sections were immersed in Tris-buffered saline (TBS) consisting of 50 mM Tris/HCl (pH 7.5) and 150 mM NaCl for 5 min at room temperature, then incubated with blocking solution containing 1% blocking reagent (Roche) in TBS for 60 min, and reacted with anti-digoxigenin Fab fragments conjugated with alkaline phosphatase (1:400 dilution; Roche) in blocking solution for 60 min at room temperature. After washing three times for 5 min with Tris-NaCl-Tween (TNT) buffer containing 50 mM Tris/HCl (pH 7.5), 150 mM NaCl and 0.05% Tween 20, the sections were rinsed in buffer comprising of 100 mM Tris/HCl (pH 9.5), 100 mM NaCl and 50 mM MgCl_2_ for 5 min. The signals were developed using 5-bromo-4-chloro-3-indolylphosphate and nitroblue tetrazolium chloride as chromogenic substrates. The slides were rinsed in Tris-EDTA buffer to stop the reaction and then mounted. The signal specificity of the mRNA for each gene was investigated in parallel experiments using the corresponding sense probes as a negative control.

### Immunohistochemistry

After the ISH procedure, the sections were washed with TNT buffer three times for 5 min, then preincubated with 1% blocking reagent (Roche) for 1 h at room temperature, and reacted with primary antibody against T1R3 (1:100; goat anti-T1R3, cat. no. sc-22458, Santa Cruz Biotechnology, Dallas, TX, USA), gustducin (1:100; rabbit anti-Gα_gust_(I-20), cat. no. sc-395, Santa Cruz Biotechnology) or Car4 (1:100; goat anti-CA4, cat. no. AF2414, R&D Systems, Minneapolis, MN, USA) in blocking reagent overnight at 4 °C. The sections were washed with TNT buffer three times for 5 min, and then reacted with appropriate secondary antibody in 1% blocking reagent: Alexa Fluor 568 donkey anti-goat IgG (Invitrogen) for T1R3, Alexa Fluor 555 donkey anti-rabbit IgG (Invitrogen) for gustducin and Alexa Fluor 568 donkey anti-goat IgG (Invitrogen) for Car4 for 2 h at room temperature. The images of the labeled taste cells were taken using the FV1000 confocal laser scanning microscope and Fluoview software (Olympus, Tokyo, Japan). We counted positively-staining cells in each taste bud in horizontal sections of the FP and CV. To exclude artifactual signals, Image-Pro Plus 4.0 (Media Cybernetics, Rockville, MD, USA) was used, that is cells showing a signal density greater than the mean plus two standard deviations of the signal density of taste cells in the negative control (primary antibody omitted) were considered positive. The same positive cells observed on contiguous sections were counted only once.

For immunohistochemical detection of Pcdh20 protein in T1R3-GFP mice, dissected tongues were fixed in 4% PFA in PBS for 45 min at 4 °C, and dehydrated with sucrose solutions (10% for 1 h, 20% for 1 h and 30% for 3 h, at 4 °C). The tongue frozen block embedded in OCT compound (Sakura Finetechnical) was sliced into serial sections (8-μm thickness), placed on silane-coated glass slides and air dried. After washing with TNT buffer, the sections were immersed in 1% blocking reagent (Roche) for 1 h at room temperature, and reacted with primary antibody against Pcdh20 (1:100; rabbit anti-Pcdh20, cat. no. bs-11113R, Bioss Inc., MA, USA) in blocking reagent overnight at 4 °C. After washing with TNT buffer three times for 5 min, sections were reacted with Alexa Fluor 647 donkey anti-rabbit IgG secondary antibody (Invitrogen) in 1% blocking reagent for 2 h at room temperature. The sections were washed with TNT buffer. The immunofluorescence of labeled taste cells was taken using FV1000 microscope and Fluoview software (Olympus).

## Supplementary information

Supplementary information.

## References

[1] Lindemann B (2001). Receptors and transduction in taste. Nature.

[2] Tu YH (2018). An evolutionarily conserved gene family encodes proton-selective ion channels. Science.

[3] Chandrashekar J, Hoon MA, Ryba NJP, Zuker CS (2006). The receptors and cells for mammalian taste. Nature.

[4] Ninomiya Y, Tonosaki K, Funakoshi M (1982). Gustatory neural response in the mouse. Brain Res..

[5] Yoshida R (2006). Taste responsiveness of fungiform taste cells with action potentials. J. Neurophysiol..

[6] Yoshida R (2009). NaCl responsive taste cells in the mouse fungiform taste buds. Neuroscience.

[7] Beidler LM, Smallman RL (1965). Renewal of cells within taste buds. J. Cell Biol..

[8] Farbman AI (1980). Renewal of taste bud cells in rat circumvallate papillae. Cell Tissue Kinet..

[9] Runge EM, Hoshino N, Biehl MJ, Ton S, Rochlin MW (2012). Neurotrophin-4 is more potent than brain-derived neurotrophic factor in promoting, attracting and suppressing geniculate ganglion neurite outgrowth. Dev. Neurosci..

[10] Treffy RW (2016). Ephrin-B/EphB signaling is required for normal innervation of lingual gustatory papillae. Dev. Neurosci..

[11] Lee H, Macpherson LJ, Parada CA, Zuker CS, Ryba NJP (2017). Rewiring the taste system. Nature.

[12] Spielman AI, Brand JG (2018). Wiring taste receptor cells to the central gustatory system. Oral Dis..

[13] De Wit J, Ghosh A (2016). Specification of synaptic connectivity by cell surface interactions. Nat. Rev. Neurosci..

[14] Takeichi M (2007). The cadherin superfamily in neuronal connections and interactions. Nat. Rev. Neurosci..

[15] Katsunuma S (2016). Synergistic action of nectins and cadherins generates the mosaic cellular pattern of the olfactory epithelium. J. Cell Biol..

[16] Duan X, Krishnaswamy A, De La Huerta I, Sanes JR (2014). Type II cadherins guide assembly of a direction-selective retinal circuit. Cell.

[17] Hondoh A (2010). Distinct expression of cold receptors (TRPM8 and TRPA1) in the rat nodose-petrosal ganglion complex. Brain Res..

[18] Chandrashekar J (2009). The taste of carbonation. Science.

[19] Adler E (2000). A novel family of mammalian taste receptors. Cell.

[20] Kim MR (2003). Regional expression patterns of taste receptors and gustducin in the mouse tongue. Biochem. Biophys. Res. Commun..

[21] Shigemura N (2008). Gurmarin sensitivity of sweet taste responses is associated with co-expression patterns of T1r2, T1r3, and gustducin. Biochem. Biophys. Res. Commun..

[22] Kusuhara Y (2013). Taste responses in mice lacking taste receptor subunit T1R1. J. Physiol..

[23] Damak S (2003). Detection of sweet and umami taste in the absence of taste receptor T1r3. Science.

[24] Thu CA (2014). Single-cell identity generated by combinatorial homophilic interactions between α, β, and γ protocadherins. Cell.

[25] Kim SY, Yasuda S, Tanaka H, Yamagata K, Kim H (2011). Non-clustered protocadherin. Cell Adh. Migr..

[26] Kim SY, Chung HS, Sun W, Kim H (2007). Spatiotemporal expression pattern of non-clustered protocadherin family members in the developing rat brain. Neuroscience.

[27] Lee W, Cheng TW, Gong Q (2008). Olfactory sensory neuron-specific and sexually dimorphic expression of protocadherin 20. J. Comp. Neurol..

[28] Vuckovic D (2015). Genome-wide association analysis on normal hearing function identifies PCDH20 and SLC28A3 as candidates for hearing function and loss. Hum. Mol. Genet..

[29] Kusakabe Y (2002). The neural differentiation gene Mash-1 has a distinct pattern of expression from the taste reception-related genes gustducin and T1R2 in the taste buds. Chem. Senses.

[30] Shigemura N (2005). Expression of amiloride-sensitive epithelial sodium channels in mouse taste cells after chorda tympani nerve crush. Chem. Senses.

[31] Yasumatsu K, Kusuhara Y, Shigemura N, Ninomiya Y (2007). Recovery of two independent sweet taste systems during regeneration of the mouse chorda tympani nerve after nerve crush. Eur. J. Neurosci..

[32] Zhang J (2019). Sour sensing from the tongue to the brain. Cell.

[33] Damak S, Mosinger B, Margolskee RF (2008). Transsynaptic transport of wheat germ agglutinin expressed in a subset of type II taste cells of transgenic mice. BMC Neurosci..

[34] Shigemura N (2004). Leptin modulates behavioral responses to sweet substances by influencing peripheral taste structures. Endocrinology.

[35] Shigemura N (2013). Angiotensin II modulates salty and sweet taste sensitivities. J. Neurosci..

[36] Shigemura, N. *et al*. Expression of renin-angiotensin system components in the taste organ of mice. *Nutrients*, **11**, 10.3390/nu11092251 (2019). 10.3390/nu11092251 PMC6770651 31546789

[37] Shigemura N, Miura H, Kusakabe Y, Hino A, Ninomiya Y (2003). Expression of leptin receptor (Ob-R) isoforms and signal transducers and activators of transcription (STATs) mRNAs in the mouse taste buds. Arch. Histol. Cytol..

[38] Shigemura N (2008). Amiloride-sensitive NaCl taste responses are associated with genetic variation of ENaC alpha-subunit in mice. Am. J. Physiol. Integr. Comp. Physiol..

