# Supplementary material for: Expression of protocadherin-20 in mouse taste buds
Source: Sci Rep. 2020 Feb 6;10:2051. doi: 10.1038/s41598-020-58991-8 (PMC7005180; doi:10.1038/s41598-020-58991-8)
Supplement: Supplementary file 1 — Supplementary information. [file 41598_2020_58991_MOESM1_ESM.pdf]

Expression of protocadherin-20 in mouse taste buds

Fumie Hirose [1,2], Shingo Takai [1], Ichiro Takahashi [2], Noriatsu Shigemura [1,3,*]

[1] Section of Oral Neuroscience, Faculty of Dental Science, Kyushu University, Fukuoka, Japan

[2] Section of Orthodontics and Dentofacial Orthopedics, Division of Oral Health, Growth and Development, Faculty of Dental Science, Kyushu University, Fukuoka, Japan

[3] Division of Sensory Physiology, Research and Development Center for Five-Sense Devices Taste and Odor Sensing, Kyushu University, Fukuoka, Japan

* Correspondence to be sent to Noriatsu Shigemura, Section of Oral Neuroscience, Graduate School of Dental Sciences, Kyushu University, 3-1-1 Maidashi, Higashi-ku, Fukuoka 812-8582, Japan. Tel. & Fax +81-92-642-6312; E-mail: shigemura@dent.kyushu-u.ac.jp

Supplementay Table S1

GeneChip data for the expression of cadherin superfamily mRNA in the taste papillae (fungiform and circumvallate papillae) and cranial ganglia (nodose-petrosal, geniculate and trigeminal ganglia).

Gene	Accession no.	FP	*Signal intensity	CV	*Signal intensity	NPG	*Signal intensity	GG	*Signal intensity	TG	*Signal intensity
Cdh1	NM_009864	4038.9	P	5618.2	P	1661.5	P	2480.9	P	2743	P
Cdh10	AF183946	15.7	A	30.2	A	531.07	P	266.27	A	416.51	P
Cdh11	NM_009866	52.2	P	57.3	A	1119.6	P	2514.1	P	1407.1	P
Cdh13	BB776961	281.9	P	298.9	P	159.64	P	597.83	P	341.08	P
Cdh15	NM_007662	49.7	A	54.1	A	125.15	A	179.52	A	224.43	A
Cdh16	NM_007663	3.5	A	26.4	A	99.63	A	131.1	A	66.667	A
Cdh17	NM_019753	17.2	A	1.7	A	15.402	A	41.539	A	28.22	A
Cdh18	BG069814	0.9	A	0.8	A	44.769	P	40.39	A	32.518	M
Cdh2	BC022107	45.6	A	85.9	P	1827.8	P	606.56	P	1736.2	P
Cdh20	NM_011800	11	A	17.5	A	197.79	P	31.966	A	204.54	P
Cdh22	AB019618	26.3	A	36.8	A	277.34	P	179.52	P	158.47	P
Cdh23	AK016015	51.8	M	26.1	A	40.294	A	71.015	A	53.629	A
Cdh26	BB748621	12.6	A	17.6	A	6.1372	A	33.607	A	27.712	A
Cdh3	X06340	33.7	A	22.7	A	122.24	A	98.349	A	151.53	P
Cdh4	NM_009867	51.2	A	93.5	P	464.61	P	288.41	M	277.72	P
Cdh5	NM_009868	15.4	A	4.4	A	772.23	P	1407.3	P	806.65	P
Cdh6	NM_007666	18.9	A	17.8	A	3225.3	P	3155.1	P	4030.7	P
Cdh7	AV327883	5.5	A	2.6	A	55.04	A	58.906	A	111.07	P
Cdh8	NM_007667	11.5	A	3.1	A	129.53	P	125.11	P	160.3	P
Cdh9	BQ176417	4	A	30.2	A	19.195	A	5.8019	A	36.497	P
Pcdh1	AK008111	81.4	P	173.3	P	60.68	A	2.0116	A	88.318	P
Pcdh10	AF334801	10.9	A	15.3	A	628.28	P	314.2	P	604.35	P
Pcdh11x	BB284627	18.5	P	3.9	A	343	P	47.283	A	348.77	P
Pcdh12	AV225493	5.6	A	7.9	A	78.836	A	67.111	A	83.162	P
Pcdh15	NM_023115	22.5	A	140.7	P	10.773	A	47.836	A	21.383	A
Pcdh17	BQ176938	11.1	A	9.6	P	2023.2	P	1463	P	994.94	P
Pcdh18	BM218630	31.9	A	52.6	A	125.41	P	217.26	P	223.5	P
Pcdh19	BB732600	484.6	P	306.1	P	292.22	P	139.6	P	435.25	P
Pcdh20	BB528056	70.4	P	531.4	P	54.782	P	185.58	P	22.358	A

Pcdh7	NM_018764	463.1	P	91.2	P	645.13	P	1018.2	P	2318.3	P
Pcdh8	BB076893	47.5	A	52.8	A	140.91	P	85.512	A	254.28	P
Pcdh9	BB244656	21	A	33.1	P	2091.3	P	2242.5	P	2962	P
Pcdha10	AB008178	11.6	A	11.7	A	90.476	A	138.7	P	113.28	P
Pcdha11	BB265776	11.2	A	32.3	A	36.907	A	13.866	A	61.922	A
Pcdhac1	BB314809	26.3	A	10.9	A	138.35	P	59.006	A	245.68	P
Pcdhb1	NM_053126	3.5	A	12.1	A	2.3526	A	6.3048	A	7.8881	A
Pcdhb10	NM_053135	9.1	A	34.5	P	118.68	A	117.68	A	148.55	P
Pcdhb11	NM_053136	8.3	A	20.4	A	14.993	A	3.316	A	41.025	A
Pcdhb12	NM_053137	2.5	A	40.7	P	78.954	P	86.372	A	57.728	P
Pcdhb13	NM_053138	15.3	A	12.3	A	135.74	A	13.353	A	131.54	A
Pcdhb14	BI734351	10.3	A	14.5	A	90.457	P	92.362	A	100.15	P
Pcdhb15	NM_053140	14.3	P	31.1	P	50.078	P	5.1835	A	117.15	P
Pcdhb16	BB027682	127.4	P	207.2	P	234.17	P	410.09	P	288.37	P
Pcdhb17	NM_053142	77.3	P	116.7	P	107.49	P	127.29	P	167.02	P
Pcdhb18	NM_053143	7.5	A	2	A	54.836	A	36.476	A	43.724	A
Pcdhb19	NM_053144	29.7	M	43.6	M	299.02	P	532.13	P	414.27	P
Pcdhb2	NM_053127	7.6	A	17.2	A	56.221	A	145.48	P	23.976	A
Pcdhb20	NM_053145	34.3	A	83.3	P	121.47	P	205.63	A	224.73	P
Pcdhb21	AV336932	53.5	M	91.5	P	107.88	A	173.58	A	143.54	P
Pcdhb22	NM_053147	24.9	P	23	P	64.265	P	67.535	M	82.957	P
Pcdhb3	NM_053128	11.8	A	29	P	42.304	A	37.81	A	103.45	P
Pcdhb4	NM_053129	4.1	A	11.5	A	1.8567	A	3.9372	A	2.2224	A
Pcdhb4	AV246533	1.9	A	13	P	42.299	P	69.796	P	26.488	P
Pcdhb5	NM_053130	23.7	A	18.3	A	9.3354	A	71.412	A	41.975	A
Pcdhb6	NM_053131	1	A	11.2	A	9.2436	A	39.978	A	22.783	A
Pcdhb7	NM_053132	10.2	P	63.6	P	32.462	A	65.588	A	73.967	P
Pcdhb8	NM_053133	5.8	A	8.9	A	21.242	A	28.438	A	59.923	A
Pcdhb9	NM_053134	19.3	P	139.6	P	168.37	P	27.883	A	171.04	P
Pcdhgc4	BG069300	30.2	A	36	P	201.34	P	155.48	A	146.32	P

Cdh, cadherin; CV, circumvallate papillae; FP, fungiform papillae; GG, geniculate ganglion; NPG, nodose-petrosal ganglion complex; Pcdh, protocadherin; TG, trigeminal ganglion. * Expression level evaluated by Affymetrix GeneChip expression analysis: P, positive expression; A, low or absent expression; M, intermediate expression between P and A levels.

Supplementay Table S2

The temporal changes in the number of Gust, T1R3 or Pcdh20 positive cells.

	Gust	T1R3	Pcdh20
P4	3±1.0	0	0
P5	6.2±1.9	0.8±0.6	0
P6	7.6±1.3	3.3±0.8	2.4±0.7
P7	9.6±1.9	5.0±1.0	3.5±0.9
P8	12.0±1.5	4.7±0.9	3.2±0.6

P, postnatal day; Gust, gustducin; T1R3, Taste receptor type 1 member 3; Pcdh20, protocadherin-20. Data are expressed as mean ± standard error of the mean per section [n=3–10 sections obtained from 1 (P4) to 4 mice (P5–8) per postnatal day, in independent preparations].

Supplementary information

Full length gel images of RT-PCR experiments (Fig 2. Protocadherin 20 (Pcdh20) mRNA is expressed in both mouse taste buds and taste ganglia, not in non-taste tongue epithelium.) Areas enclosed by red dot line were used in Fig 2.

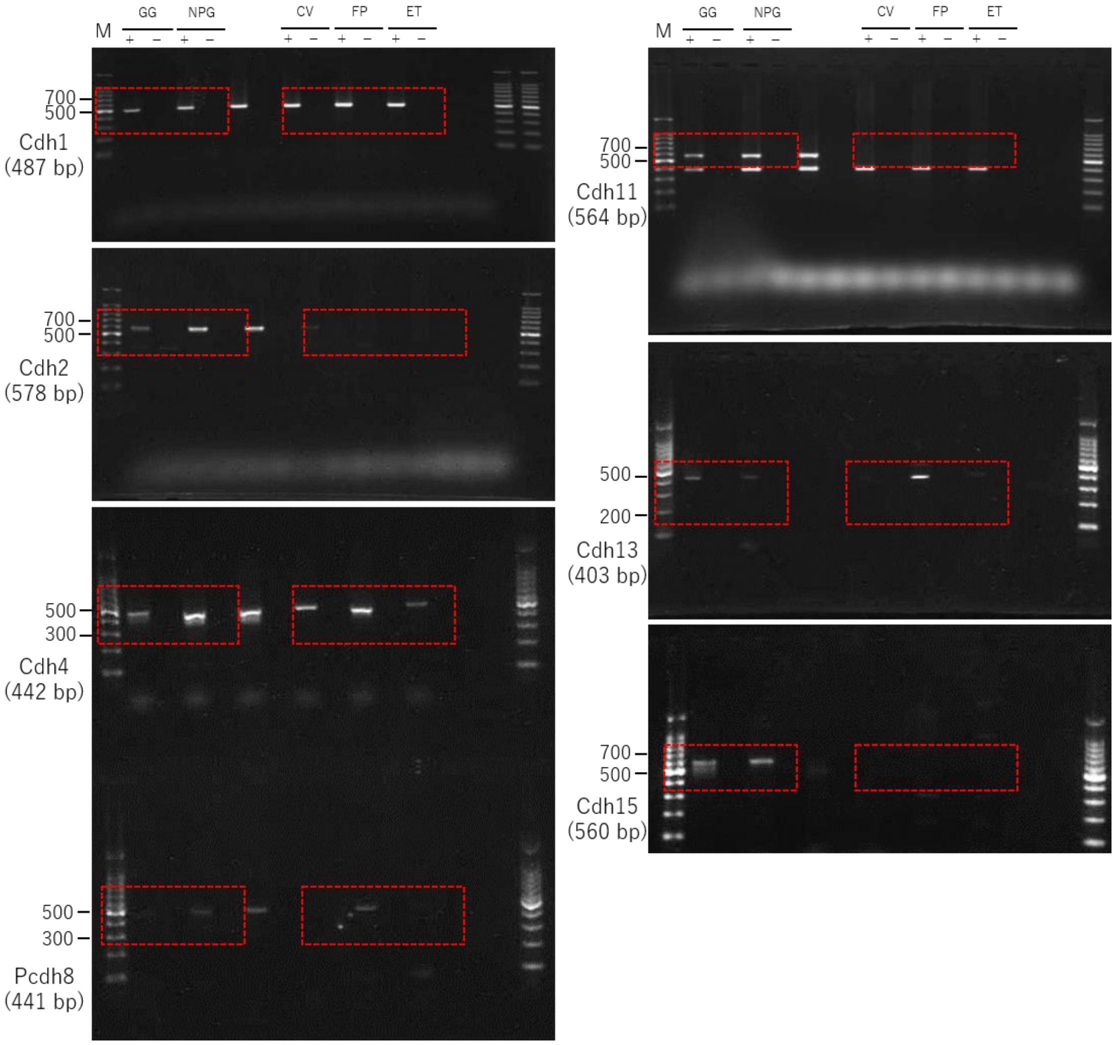

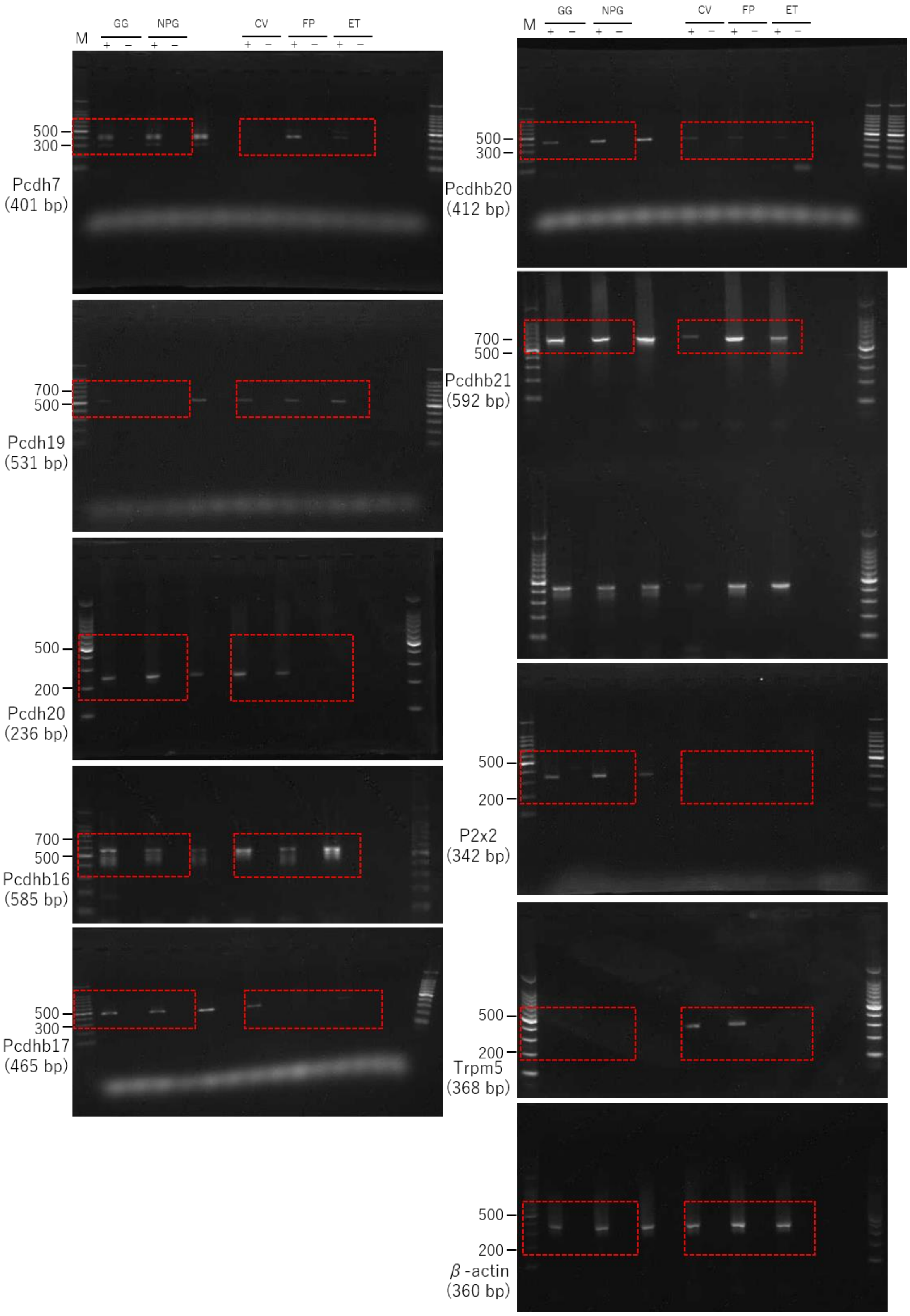
M
GG
NPG
CV
FP
ET
+ − + − + − + − + −
500
300
Pcdh7
(401 bp)
700
500
Pcdh19
(531 bp)
500
200
Pcdh20
(236 bp)
700
500
Pcdhb16
(585 bp)
500
300
Pcdhb17
(465 bp)
M
GG
NPG
CV
FP
ET
+ − + − + − + − + −
500
300
Pcdhb20
(412 bp)
700
500
Pcdhb21
(592 bp)
500
200
P2x2
(342 bp)
500
200
Trpm5
(368 bp)
500
200
β-actin
(360 bp)
